# Molecular Mechanisms Involved in Mesenchymal Stem Cell Migration to the Site of Acute Myocardial Infarction

**DOI:** 10.1155/2009/904682

**Published:** 2009-07-12

**Authors:** Katarina Kollar, Matthew M. Cook, Kerry Atkinson, Gary Brooke

**Affiliations:** ^1^Adult Stem Cell Laboratory, Mater Medical Research Institute, South Brisbane, QLD 4101, Australia; ^2^School of Medicine, University of Queensland, St. Lucia, QLD 4072, Australia

## Abstract

Mesenchymal stem cells or multipotent mesenchymal stromal cells (both referred to as MSC) have been shown in some studies to have a beneficial effect on myocardial recovery after infarct. Current strategies for MSC delivery to heart involve intravenous, intraarterial, and intramuscular delivery. Different routes of MSC delivery and a lack of knowledge of the mechanisms that MSC utilise to migrate in vivo has most likely led to the marked variations in results that have been found. This review aims to summarise the current knowledge of MSC migratory mechanisms and looks to future methods of MSC manipulation prior to delivery in order to enhance MSC migration and engraftment.

## 1. Introduction

Cardiovascular disease is currently a leading cause of death worldwide [1] indicating that global primary prevention and effective secondary treatments are urgently needed. Advances have been made in the treatment and management for cardiovascular disease but despite this, cannot directly reverse the disease process, that is, replace lost cardiomyocytes and/or the myocardial scar with new fully functional myocardial cells.

MSC are multipotent cells that are capable of differentiating into cells of the mesodermal lineage. In vivo, MSC are present as a rare population in the bone marrow and possibly other tissues such as placenta, adipose tissue, and blood vessels (as perivascular cells) [2–5]. MSC are expanded in vitro before use and thus the properties attributed to MSC are of these ex vivo expanded cells. MSC also have highly immunosuppressive properties [4] and there is evidence that ex vivo expanded MSC can engraft within tissues in many settings including myocardial damage after myocardial infarction (AMI) [6].

## 2. Mesenchymal Stem Cells

MSC were first described by Friedenstein et al. [7] as an adherent, fibroblast-like population that could regenerate rudiments of normal bone in vivo [7–9]. MSC are located within the stroma of the bone marrow and represent ~0.0001% of nucleated bone marrow cells [10, 11]. When isolated from various tissues [12–15] and expanded ex vivo, these cells have been shown to differentiate into cell types of mesenchymal lineage, including bone, cartilage, muscle, adipose tissue, and bone marrow stroma [10, 16, 17]. Until recently, MSC had not been shown to be true stem cells, that is, cells capable of serial transfer between animals with the ability to reconstitute a fully functioning tissue of origin. However, two groups have recently shown that ex vivo expanded MSC are capable of such behaviour [14, 18, 19]. Ex vivo expanded MSC have been characterised by flow cytometry with a variety of markers. Some of these, including CD73, CD90, and CD105 [10, 20, 21] are indicative (but not definitively so) of MSC phenotype. Also, MSC do not express typical haematopoietic antigens including CD45, CD34, and CD14 [22].

MSC are an attractive cellular therapeutic candidate due to their relative ease of isolation using standard culture media with bovine serum [30]. In the murine system contaminating haematopoietic cells are not readily lost using the standard adherence protocol [20], but enrichment of mouse MSC can be achieved by using flow cytometry to selection cells based on Sca-1^+^, CD45^-^ [31]. A lack of definitive phenotypic properties and isolation techniques, especially for murine MSC may have made it difficult to compare MSC derived from different laboratories. Several investigators have tried to resolve this problem and several antibodies have been utilised to facilitate the prospective isolation of MSC such as the STRO-1 mAb [32]. Recently, Battula et al. [33] described a panel of monoclonal antibodies with superior selectivity for MSC, including the monoclonal antibodies W8B2 against human mesenchymal stem cell antigen-1 (MSCA-1) and CD56. CFU-F assays showed that MSC can be enriched with MSCA-1 and CD56 and have the ability to differentiate into mesodermal lineage. Selection of MSC using nerve growth factor receptor (NGFR) antibodies may also be used [34–36]. NGFR has also been described on the earliest component of BM stroma in developing human foetal epiphyseal bone [37, 38] and in a small percentage of cells from the adherent layer of BM cultures, thus suggesting that NGFR antibodies also may stain primitive MSC. However, the use of a definitively phenotyped MSC population remains an unmet goal in MSC research.

## 3. MSC Engraftment in AMI

In the laboratory, animal models of AMI have been widely used to study therapies aimed at improving the recovery from ischaemic organ damage. Several preclinical studies and clinical trials have reported that MSC attenuate maladaptive left ventricular (LV) remodeling, and preserve and/or promote recovery of pump performance after myocardial infarction [39–41]. The mechanism underpinning these effects has been variously attributed to de novo cardiomyogenesis, and/or neoangiogenesis [40]. A growing body of evidence suggests, however, that the therapeutic effects of MSC transplantation primarily result from indirect stimulation (often termed paracrine) of neovascularisation and protection from ischemia-induced apoptosis [40, 41].

Intramyocardial injection has been the most widely used delivery route for transplanting MSC into infarcted myocardium [42]. Although this technique guarantees localised delivery to the inflamed tissue, it has restricted clinical applicability because it is invasive, and can lead to cardiac arrhythmias. Systemic delivery of MSC provides a minimally invasive and clinically acceptable alternative, and has been investigated using various animal models of myocardial infarction [43–47]. The major problem with this delivery route seems to be loss of MSC in the vasculature, mostly in the lungs and liver and generally only a small proportion of injected MSC are found in the ischaemic damaged myocardium [43, 46]. Despite this, Nagaya et al. [44], showed that intravenous (IV) infusion of MSC 3 hours after AMI resulted in a reduced infarct size and a slight improvement in LV function one month later. After 24 hours, approximately 3% of injected MSC were found engrafted, mostly in the border zone of infarcted myocardium. Similarly, Jiang et al. [45] demonstrated that systemic infusion of MSC 3 hours after AMI resulted in migration to inflamed myocardium, and produced a reduction in infarct size and improvements in LV function. These results confirmed the findings of Boomsma et al. [47] who in a mouse model of AMI delivered MSC 1 hour after coronary artery occlusion, and demonstrated functional improvements relative to vehicle-injected controls at 14 days. Cheng et al. [42] demonstrated significant cell engraftment in rats receiving MSC infusion 24 hours after AMI, with efficiency of engrafted MSC doubled, relative when CXCR4-overexpressing MSC were used. While they found no effect on infarct size, MSC were shown to attenuate postinfarct systolic function. Two studies have directly compared intracoronary and intravenous routes of MSC delivery after AMI [43, 49]. While both found that the majority of cells were trapped in the lung regardless of delivery route, higher numbers of MSC were observed in the peri-infarct zone after intracoronary injection. Neither study measured the effect of this improved engraftment on organ function. Entrapment of cells in the lungs is possibly due to their relatively large size of ex vivo expanded MSC. However, others have shown that MSC are capable of efficient migration to injured tissues after IV delivery [44, 50]. This suggests that MSC expresses specific receptors or ligands to facilitate trafficking, adhesion, and infiltration to sites of injury.

## 4. Migratory Mechanisms Utilised by MSC

**Figure 1:**
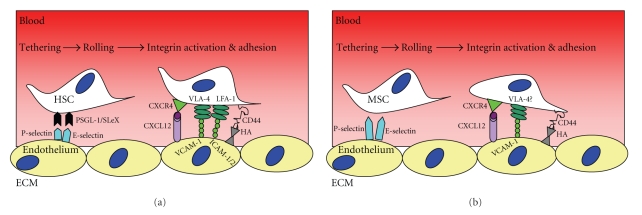
Schematic illustration of the molecular mechanisms used by (a) leukocytes for homing to the bone marrow and (b) differences with MSC.

The well-documented migration of leukocytes into inflamed tissues is probably a good paradigm to compare MSC migration from the blood stream into inflamed tissues. The capillary endothelium (excluding bone marrow) does not constitutively express E-, L-, and P-selectins [48]. However, upon activation, these cell adhesion molecules are rapidly presented at the surface of endothelium, initiating leukocyte rolling. Chemokines released during inflammation induce firm adhesion and chemoattraction of leukocytes, and the type of chemokine released can determine the subtypes of leukocytes that migrate [51, 52]. Integrins are normally in a low affinity state, but can be switched to a high affinity state by the action of chemokines [53]. Firm adhesion is followed by diapedesis across endothelial tight junctions and basement membrane allowing movement into the extracellular matrix (ECM) of the local tissue stroma. Here cells adhere to ECM components such as hyaluronic acid, laminin, collagen, and fibronectin via integrins, CD44, and other cell adhesion molecules. Migration through the ECM is facilitated by ECM-degrading enzymes such as the matrix metalloproteases (MMPs) which free bound chemokines allow movement along the chemokine gradient within the local tissue (Figure 1). Although this process represents a likely paradigm for MSC migration, all of the molecular mediators and chemotactic signals that guide MSC to appropriate microenvironments are yet to be fully identified [29].

## 5. Role of Chemokines in Acutely Infarcted Myocardium

Chemokines comprise a family of small (8–14 kDa) highly basic proteins with similar tertiary structure that play a critical role in basal and inflammatory leukocyte locomotion and trafficking [54–56]. In addition to effects on cell locomotion, certain chemokines are capable of eliciting a variety of other responses affecting leukocyte adhesion, activation, degranulation, mitogenesis, and apoptosis. Furthermore, chemokines have a wide range of effects on many different cell types beyond the immune system, including endothelial cells, fibroblasts, smooth muscle cells, neurons, and epithelial cells [57–59]. Chemokine induction is an important mechanistic response to myocardial injury during inflammation. In myocardial infarcts, cellular necrosis triggers several chemokine-inducing pathways which are regulated through free radical generation, nuclear factor-*κ*B activation, TNF-*α* release, and complement activation [60, 61].

Studies using animal models of AMI have demonstrated that many chemokines are up-regulated in the infarcted heart and suggest they play a role in regulating postinfarction inflammatory response [62]. Chemokines released after myocardial ischaemia include CCL2 (MCP-1), CCL3 (MIP-1*α*/), CCL4 (MIP-1*β*), CXCL8 (IL-8), CXCL10 (IP-10), and CXCL12 (SDF-1) [60]. Chemokine receptors, including CXCR4, which are known to be involved in the migration of leukocytes across the endothelium have been reported to be expressed on MSC, however, this expression appears to be variable (Table 2). This may be due to regulation of chemokine receptors through internalisation from the cell surface and degradation within the cytoplasm. It may also be a feature of ex vivo highly expanded cells [63, 64].

Freshly isolated MSC express surface CXCR4 and this has been suggested to be of importance for MSC migration [65]. However, some have shown that CXCR4 expression is markedly reduced during ex vivo expansion leading to decreased migration of the cells towards CXCL12 [65, 66]. Furthermore it has been shown that CXCR4 surface receptor expression was present on few if any ex vivo cultured human MSC, although intracellular CXCR4 expression could be detected [29, 67]. The importance of CXCR4 in MSC migration has been questioned [68]. The study by Ip et al. [68] suggested that MSCs do not require CXCR4 for myocardial migration and engraftment. It should be noted that this study utilised direct intramyocardial injection rather than intravenous delivery [68]. Therefore, it may be the lack of surface expression of CXCR4 by MSC that leads to low efficiency of migration towards CXCL12. This has been further supported by the observation that enforced surface expression of CXCR4 leads to increased MSC engraftment and functional recovery after AMI [49, 69, 70]. Shi et al. [65] investigated induced upregulation of CXCR4 in migration of Flk1^+^ MSC derived from human fetal bone marrow. They showed that CXCR4 expression can be rapidly induced on the cell surface after stimulation with the cytokines Flt-3 ligand, stem cell factor (SCF), interleukin (IL)-6, hepatocyte growth factor (HGF), and IL-3. Upregulation of CXCR4 increased in vivo migration capacity to CXCL12 and homing to the bone marrow of irradiated NOD/SCID mice. Finally, we would like to suggest that a further confounder to CXCR4 mediated migration of MSC is that MSC constitutively produce CXCL12 (which may be a reflection of their previous bone marrow stromal role). Although it is assumed that the endogenous CXCL12 is not affecting MSC migration [71], it may be possible that it actually interferes both with their surface expression of CXCR4 and with their migration. Hence, at least in terms of CXCL12-mediated migration of MSC, ex vivo manipulated MSC maybe more suitable for the treatment where intravenous delivery and migration of MSC is required.

## 6. Adhesion Molecules and Signalling Pathways

The molecular mechanism underlying migration of cells from the blood stream involve a complex multistep process required to cope with shear forces associated with transendothelial migration. MSC have been shown to express various adhesion molecules including CD106 (VCAM-1), CD54 (ICAM-1), CD50 (ICAM-3), CD166 (ALCAM), CD44, and integrins including *α*1, *α*2, *α*3, *α*4, *α*5, *α*v, *β*1, *β*3, and *β*4, many of which are thought to be involved in migration (Table 1) [72].

Selectin receptors are required for initial rolling in the capillaries and consist of glyoproteins with fucoslyated glycan side chains, for example, PSGL-1, CD34 [73, 74]. Cells cannot adhere to selectins without the coexpression of glycosyl-transferases required to generate sialyl Lewis-X (sLe^X^) core-2 O-glycans (reviewed [74]). In particular, fucosyl transferase (FUT)-7 is essential to generate functional selectin receptors as its loss leads to inactivation of P- and E-selectin receptors [75]. There has been some evidence to support selectin-mediated adhesion by MSC [76], but others have shown that MSC do not express FUT-4 or FUT-7 and do not functionally bind to E- or P-selectin in vitro, indicating that no selectin binding by MSC is possible [29]. Furthermore, artificial enzymatic addition of sLex on MSC using recombinant FUT6 has been shown to induce selectin binding and increased homing of MSC to the bone marrow [77].

Firm adhesion, which follows rolling in capillaries is achieved via integrins. However, this may be limited in MSC (especially BM-derived MSC), which have low levels of VLA-4 (CD49d) and murine MSC do not express VLA-4 at all [29]. Thus interaction with VCAM-1 on endothelium (allowing firm adhesion) does not appear to be possible. Therefore, the exact manner of MSC firm adhesion to endothelium (and therefore emigration from capillaries) remains unclear but it is possible that high expression of CD44 by MSC may allow sufficient adhesion to the endothelium [78]. Herrera et al. [79] investigated how interactions between CD44 and HA influence exogenous MSC migration to the kidneys after acute renal failure. These cells were isolated from wild-type or CD44-null mice. The data showed that the expression of CD44 was important for MSC migration to inflamed tissue. MSC that have had CD44 blocked by a neutralizing antibody also had reduced capacity to reach the damaged kidneys. Furthermore, in vitro studies supported the involvement of CD44 in chemotaxis toward purified HA, as CD44-null MSC or MSC transfected to express a defective variant of CD44 did not migrate.

The role of MMPs has also been studied in MSC migration. When matrix metalloproteinase-2 (MMP-2) was blocked with a neutralising antibody, in vitro transendothelial migration was impaired [80]. De Becker et al. [80] also showed that blocking MMP-2 with an inhibitory antibody or siRNA leads to increased expression of tissue inhibitor metalloproteinase-3 (TIMP-3). Interestingly, the migratory capacity of MSC was strongly affected in both studies by the level of culture confluence, where significant decrease of migration occurred for cells cultured at higher concentrations. MMP-2 can be activated at the cell surface by membrane-type matrix metalloproteinase-1 (MT1-MMP) [81]. This activation has been shown to be important in human MSC invasion through basement membranes, where MT1-MMP was upregulated upon activation of Wnt signalling. Wnt signalling regulates other basic stem cell features, such as self-renewal of intestinal epithelial stem cell [82] or haematopoietic stem cells [83].

Considerable research has been devoted to the effectors of stem cell migration and engraftment. However, rather less attention has been paid to the signal transduction pathways eliciting these mechanisms. In particular, the molecular signalling cascades governing MSC migration is of major importance, with the Wnt signalling pathway being associated with migration and invasion [84]. The activation of the Wnt signal transduction pathway by Wnt3a-conditioned medium was shown to stimulate MSC proliferation, while retaining pluriotency [85]. Neth et al. utilised siRNA to knock down expression of *β*-catenin, a transcriptional activator for the Wnt signalling pathway, which resulted in down-regulation of Wnt target genes cyclin D1 and MT1-MMP. Reduced proliferation rate and diminished invasive capacity of MSC were observed, showing that Wnt signalling is involved in MSC migration.

Several growth factors (GFs) have also been reported to be involved in MSC migration. One such GF involved in MSC (and epithelial cell) migration is Hepatocyte growth factor (HGF) [86]. Studies have demonstrated that human MSCs constitutively express the HGF ligand c-met and can migrate in response to HGF [86]. Thus, controlled activation of Wnt signalling or HGF ligand may enhance MSC migration and invasion when tissue regeneration is needed but on the other hand it may negatively affect characteristic stem cell features such as self-renewal.

## 7. Conclusions

The ability of MSC to specifically migrate to and engraft in injured tissue is still under intense scrutiny. Migration studies of MSC to sites of acute myocardial infarction have highlighted the importance of chemokines, however, specific mechanisms remain poorly understood. Substantial challenges remain in order to optimise MSC migration and fulfill its promise as an easily intravenously delivered therapeutic in clinical practice. Until in vivo MSC phenotype with respect to migratory molecules can be controlled and/or standardised, inconsistent results will persist. Basic research is still required to optimise the techniques by which these cells are isolated, cultured, and manipulated in vitro to improve their migration, engraftment, survival, and function following administration to patients after severe acute myocardial infarction.

## Figures and Tables

**Table 1:** Characteristics of bone marrow-derived MSC: expression of specific antigens, cytokine receptors and adhesion molecules, as well as production of cytokines and matrix molecules.

Expression on MSC	Designation	Reference
Characteristic cell surface antigens	CD73, CD90, CD105, CD166, STRO-1	[17, 22–24]
Cytokines and growth factors	IL- 1*α*, IL-1*β*, IL-6, IL-7, IL-8, IL-11, IL-12, IL-14, and IL-15 LIF, SCF, Flt-3 ligand, GM-CSF, G-CSF, M-CSF, VEGF	[17, 25]
Cytokine receptors and growth factor receptors	IL-1R (CD121), IL-2R (CD25), IL-3R (CD123), IL-4R (CD124), IL-6R (CD126), IL-7R (CD127), LIFR, SCFR, G-CSFR, IFN*γ*R, TNFIR, TNFIIR, TGF*β*IR, TGF*β*IIR, bFGFR, PDGFR, EGFR	[10, 22, 26, 27]
Adhesion	Integrins: *α*1, *α*2, *α*3, *α*4, *α*5, *α*v, *β*1, *β*3, *β*4, ICAM-1 (CD54), ICAM-2 (CD102), VCAM-1 (CD106), ALCAM-1 (CD166), LFA-3 (CD58), L-selectin (CD62L), endoglin (CD105), CD44, VLA-4	[10, 22, 28, 29]

**Table 2:** Expression of chemokine receptor mRNA by human and murine bone marrow derived MSC as determined by microarray or qRT-PCR and flow cytometry.

Chemokine receptor	mRNA		Cell surface protein		Intracellular protein	
	Human	Mouse	Human	Mouse	Human	Mouse
CCR1	−	+	+/−	ND	+	ND
CCR2	−	−		−		−
CCR3	−	−	−	−	+	−
CCR4	−	−	ND	ND	ND	ND
CCR5	+	+	−	−	−	+
CCR6	−	−	ND	ND	ND	ND
CCR7	−	+	ND	−	ND	+
CCR8	+	+	+/−	−	−	ND
CCR9	−	+	ND	−	ND	ND
CCR10	+	+	−	−	+/−	ND
CCR11	+	+	+/−	ND	−	ND
CXCR1	−	−	ND	ND	ND	ND
CXCR2	−	−	ND	ND	ND	ND
CXCR3	−	−	−	ND	+	ND
CXCR4	−	+	−	−	+	+
CXCR5	−	−		+	ND	ND
CXCR6	−	+	+	+	+	ND
CXCR7	ND	−	ND	ND	ND	ND
CX3CR1	−	−	ND	ND	ND	ND

−, not detected; +/−, weak expression, +, strong expression; ND, no data

## References

[1] Bonow RO, Smaha LA, Smith SC, Mensah GA, Lenfant C (2002). World Heart Day 2002: the international burden of cardiovascular disease: responding to the emerging global epidemic. *Circulation*.

[2] Barlow S, Brooke G, Chatterjee K (2008). Comparison of human placenta- and bone marrow-derived multipotent mesenchymal stem cells. *Stem Cells and Development*.

[3] Brooke G, Rossetti T, Pelekanos R (2009). Manufacturing of human placenta-derived mesenchymal stem cells for clinical trials. *British Journal of Haematology*.

[4] Yañez R, Lamana ML, García-Castro J, Colmenero I, Ramírez M, Bueren JA (2006). Adipose tissue-derived mesenchymal stem cells have in vivo immunosuppressive properties applicable for the control of the graft-versus-host disease. *Stem Cells*.

[5] Crisan M, Yap S, Casteilla L (2008). A perivascular origin for mesenchymal stem cells in multiple human
organs. *Cell Stem Cell*.

[6] Orlic D, Kajstura J, Chimenti S (2001). Mobilized bone marrow cells repair the infarcted heart, improving function and survival. *Proceedings of the National Academy of Sciences of the United States of America*.

[7] Friedenstein AJ, Petrakova KV, Kurolesova AI, Frolova GP (1968). Heterotopic of bone marrow. Analysis of precursor cells for osteogenic and hematopoietic tissues. *Transplantation*.

[8] Friedenstein AJ (1976). Precursor cells of mechanocytes. *International Review of Cytology*.

[9] Owen M, Friedenstein AJ (1988). Stromal stem cells: marrow-derived osteogenic precursors. *Ciba Foundation Symposium*.

[10] Pittenger MF, Mackay AM, Beck SC (1999). Multilineage potential of adult human mesenchymal stem cells. *Science*.

[11] Gronthos S, Zannettino ACW, Hay SJ (2003). Molecular and cellular characterisation of highly purified stromal stem cells derived from human bone marrow. *Journal of Cell Science*.

[12] De Ugarte DA, Morizono K, Elbarbary A (2003). Comparison of multi-lineage cells from human adipose tissue and bone marrow. *Cells Tissues Organs*.

[13] Campagnoli C, Roberts IAG, Kumar S, Bennett PR, Bellantuono I, Fisk NM (2001). Identification of mesenchymal stem/progenitor cells in human first-trimester fetal blood, liver, and bone marrow. *Blood*.

[14] Noort WA, Kruisselbrink AB, In't Anker PS (2002). Mesenchymal stem cells promote engraftment of human umbilical cord blood-derived CD34^+^ cells in NOD/SCID mice. *Experimental Hematology*.

[15] Erices A, Conget P, Minguell JJ (2000). Mesenchymal progenitor cells in human umbilical cord blood. *British Journal of Haematology*.

[16] Prockop DJ (1997). Marrow stromal cells as stem cells for nonhematopoietic tissues. *Science*.

[17] Haynesworth SE, Baber MA, Caplan AI (1996). Cytokine expression by human marrow-derived mesenchymal progenitor cells in vitro: effects of dexamethasone and IL-1*α*. *Journal of Cellular Physiology*.

[18] Sacchetti B, Funari A, Michienzi S (2007). Self-renewing osteoprogenitors in bone marrow sinusoids can
organize a *hematopoietic* microenvironment. *Cell*.

[19] Belema-Bedada F, Uchida S, Martire A, Kostin S, Braun T (2008). Efficient homing of multipotent adult mesenchymal stem cells
depends on FROUNT-mediated clustering of CCR2. *Cell Stem Cell*.

[20] Javazon EH, Tebbets J, Beggs K (2003). Isolation, expansion, and characterization of murine adult bone marrow derived, mesenchymal stem cells. *Blood*.

[21] Peister A, Mellad JA, Larson BL, Hall BM, Gibson LF, Prockop DJ (2004). Adult stem cells from bone marrow (MSCs) isolated from different strains of inbred mice vary in surface epitopes, rates of proliferation, and differentiation potential. *Blood*.

[22] Conget PA, Minguell JJ (1999). Phenotypical and functional properties of human bone marrow mesenchymal progenitor cells. *Journal of Cellular Physiology*.

[23] Galmiche MC, Koteliansky VE, Briere J, Herve P, Charbord P (1993). Stromal cells from human long-term marrow cultures are mesenchymal cells that differentiate following a vascular smooth muscle differentiation pathway. *Blood*.

[24] Simmons PJ, Torok-Storb B (1991). Identification of stromal cell precursors in human bone marrow by a novel monoclonal antibody, STRO-1. *Blood*.

[25] Majumdar MK, Thiede MA, Mosca JD, Moorman M, Gerson SL (1998). Phenotypic and functional comparison of cultures of marrow-derived mesenchymal stem cells (MSCs) and stromal cells. *Journal of Cellular Physiology*.

[26] Gronthos S, Simmons PJ (1995). The growth factor requirements of STRO-1-positive human bone marrow stromal precursors under serum-deprived conditions in vitro. *Blood*.

[27] Satomura K, Derubeis AR, Fedarko NS (1998). Receptor tyrosine kinase expression in human bone marrow stromal cells. *Journal of Cellular Physiology*.

[28] Bruder SP, Ricalton NS, Boynton RE (1998). Mesenchymal stem cell surface antigen SB-10 corresponds to activated leukocyte cell adhesion molecule and is involved in osteogenic differentiation. *Journal of Bone and Mineral Research*.

[29] Brooke G, Tong H, Levesque J-P, Atkinson K (2008). Molecular trafficking mechanisms of multipotent mesenchymal stem cells derived from human bone marrow and placenta. *Stem Cells and Development*.

[30] Shahdadfar A, Frønsdal K, Haug T, Reinholt FP, Brinchmann JE (2005). In vitro expansion of human mesenchymal stem cells: choice of serum is a determinant of cell proliferation, differentiation, gene expression, and transcriptome stability. *Stem Cells*.

[31] Haynesworth SE, Baber MA, Caplan AI (1992). Cell surface antigens on human marrow-derived mesenchymal cells are detected by monoclonal antibodies. *Bone*.

[32] Rombouts WJC, Ploemacher RE (2003). Primary murine MSC show highly efficient homing to the bone marrow but lose homing ability following culture. *Leukemia*.

[33] Battula VL, Treml S, Bareiss PM (2009). Isolation of functionally distinct mesenchymal stem cell subsets using antibodies against CD56, CD271, and mesenchymal stem cell antigen-1. *Haematologica*.

[34] Thomson TM, Rettig WJ, Chesa PG, Green SH, Mena AC, Old LJ (1988). Expression of human nerve growth factor receptor on cells derived from all three germ layers. *Experimental Cell Research*.

[35] Chesa PG, Rettig WJ, Thomson TM, Old LJ, Melamed MR (1988). Immunohistochemical analysis of nerve growth factor receptor expression in normal and malignant human tissues. *Journal of Histochemistry and Cytochemistry*.

[36] Thompson SJ, Schatteman GC, Gown AM, Bothwell M (1989). A monoclonal antibody against nerve growth factor receptor. Immunohistochemical analysis of normal and neoplastic human tissue. *American Journal of Clinical Pathology*.

[37] Weiss L, Chen LT (1975). The organization of hematopoietic cords and vascular sinuses in bone marrow. *Blood Cells*.

[38] Chen LT, Weiss L (1975). The development of vertebral bone marrow of human fetuses. *Blood*.

[39] Abdel-Latif A, Bolli R, Tleyjeh IM (2007). Adult bone marrow-derived cells for cardiac repair: a systematic review and meta-analysis. *Archives of Internal Medicine*.

[40] Laflamme MA, Zbinden S, Epstein SE, Murry CE (2007). Cell-based therapy for myocardial ischemia and infarction: pathophysiological mechanisms. *Annual Review of Pathology*.

[41] Segers VFM, Lee RT (2008). Stem-cell therapy for cardiac disease. *Nature*.

[42] Cheng Z, Ou L, Zhou X (2008). Targeted migration of mesenchymal stem cells modified with CXCR4 gene to infarcted myocardium improves cardiac performance. *Molecular Therapy*.

[43] Barbash IM, Chouraqui P, Baron J (2003). Systemic delivery of bone marrow-derived mesenchymal stem cells to the infarcted myocardium: feasibility, cell migration, and body distribution. *Circulation*.

[44] Nagaya N, Fujii T, Iwase T (2004). Intravenous administration of mesenchymal stem cells improves cardiac function in rats with acute myocardial infarction through angiogenesis and myogenesis. *American Journal of Physiology*.

[45] Jiang W, Ma A, Wang T (2006). Homing and differentiation of mesenchymal stem cells delivered intravenously to ischemic myocardium in vivo: a time-series study. *Pflugers Archiv*.

[46] Price MJ, Chou C-C, Frantzen M (2006). Intravenous mesenchymal stem cell therapy early after reperfused acute myocardial infarction improves left ventricular function and alters electrophysiologic properties. *International Journal of Cardiology*.

[47] Boomsma RA, Swaminathan PD, Geenen DL (2007). Intravenously injected mesenchymal stem cells home to viable myocardium after coronary occlusion and preserve systolic function without altering infarct size. *International Journal of Cardiology*.

[48] Schweitzer KM, Dräger AM, van der Valk P (1996). Constitutive expression of E-selectin and vascular cell adhesion molecule-1 on endothelial cells of *hematopoietic* tissues. *The American Journal of Pathology*.

[49] Freyman T, Polin G, Osman H (2006). A quantitative, randomized study evaluating three methods of mesenchymal stem cell delivery following myocardial infarction. *European Heart Journal*.

[50] Kraitchman DL, Tatsumi M, Gilson WD (2005). Dynamic imaging of allogeneic mesenchymal stem cells trafficking to myocardial infarction. *Circulation*.

[51] Ma J, Ge J, Zhang S (2005). Time course of myocardial stromal cell-derived factor 1 expression and beneficial effects of intravenously administered bone marrow stem cells in rats with experimental myocardial infarction. *Basic Research in Cardiology*.

[52] Ringe J, Strassburg S, Neumann K (2007). Towards in situ tissue repair: human mesenchymal stem cells express chemokine receptors CXCR1, CXCR2 and CCR2, and migrate upon stimulation with CXCL8 but not CCL2. *Journal of Cellular Biochemistry*.

[53] Hood JD, Cheresh DA (2002). Role of integrins in cell invasion and migration. *Nature Reviews Cancer*.

[54] Clark-Lewis I, Kim K-S, Rajarathnam K (1995). Structure-activity relationships of chemokines. *Journal of Leukocyte Biology*.

[55] Gerard C, Rollins BJ (2001). Chemokines and disease. *Nature Immunology*.

[56] Moser B, Loetscher P (2001). Lymphocyte traffic control by chemokines. *Nature Immunology*.

[57] Baggiolini M (2001). Chemokines in pathology and medicine. *Journal of Internal Medicine*.

[58] Gerszten RE, Garcia-Zepeda EA, Lim Y-C (1999). MCP-1 and IL-8 trigger firm adhesion of monocytes to vascular endothelium under flow conditions. *Nature*.

[59] Strieter RM, Polverini PJ, Arenberg DA (1995). Role of C-X-C chemokines as regulators of angiogenesis in lung cancer. *Journal of Leukocyte Biology*.

[60] Frangogiannis NG, Smith CW, Entman ML (2002). The inflammatory response in myocardial infarction. *Cardiovascular Research*.

[61] Dewald O, Ren G, Duerr GD (2004). Of mice and dogs: species-specific differences in the inflammatory
response following myocardial infarction. *The American Journal of Pathology*.

[62] Dobaczewski M, Frangogiannis NG (2008). Chemokines in myocardial infarction: translating basic research into clinical medicine. *Future Cardiology*.

[63] Dar A, Goichberg P, Shinder V (2005). Chemokine receptor CXCR4-dependent internalization and resecretion of functional chemokine SDF-1 by bone marrow endothelial and stromal cells. *Nature Immunology*.

[64] Honczarenko M, Le Y, Swierkowski M, Ghiran I, Glodek AM, Silberstein LE (2006). Human bone marrow stromal cells express a distinct set of biologically functional chemokine receptors. *Stem Cells*.

[65] Shi M, Li J, Liao L (2007). Regulation of CXCR4 expression in human mesenchymal stem cells by cytokine treatment: role in homing efficiency in NOD/SCID mice. *Haematologica*.

[66] Ponte AL, Marais E, Gallay N (2007). The in vitro migration capacity of human bone marrow mesenchymal stem cells: comparison of chemokine and growth factor chemotactic activities. *Stem Cells*.

[67] Wynn RF, Hart CA, Corradi-Perini C (2004). A small proportion of mesenchymal stem cells strongly expresses functionally active CXCR4 receptor capable of promoting migration to bone marrow. *Blood*.

[68] Ip JE, Wu Y, Huang J, Zhang L, Pratt RE, Dzau VJ (2007). Mesenchymal stem cells use integrin *β*1 not CXC chemokine receptor 4 for myocardial migration and engraftment. *Molecular Biology of the Cell*.

[69] Ryser MF, Ugarte F, Thieme S, Bornhäuser M, Roesen-Wolff A, Brenner S (2008). mRNA transfection of CXCR4-GFP fusion—simply generated by PCR—results in efficient migration of primary human mesenchymal stem cells. *Tissue Engineering. Part C*.

[70] Cheng Z, Liu X, Ou L (2008). Mobilization of mesenchymal stem cells by granulocyte colony-stimulating factor in rats with acute myocardial infarction. *Cardiovascular Drugs and Therapy*.

[71] Beider K, Abraham M, Peled A (2008). Chemokines and chemokine receptors in stem cell circulation. *Frontiers in Bioscience*.

[72] Minguell JJ, Erices A, Conget P (2001). Mesenchymal stem cells. *Experimental Biology and Medicine*.

[73] Wagers AJ, Lowe JB, Kansas GS (1996). An important role for the *α*1,3 fucosyltransferase, FucT-VII, in leukocyte adhesion to E-selectin. *Blood*.

[74] Lowe JB (2002). Glycosylation in the control of selectin counter-receptor structure and function. *Immunological Reviews*.

[75] Malý P, Thall AD, Petryniak B (1996). The *α*(1,3)fucosyltransferase Fuc-TVII controls leukocyte trafficking through an essential role in L-, E-, and P-selectin ligand biosynthesis. *Cell*.

[76] Rüster B, Göttig S, Ludwig RJ (2006). Mesenchymal stem cells display coordinated rolling and adhesion behavior on endothelial cells. *Blood*.

[77] Xia L, McDaniel JM, Yago T, Doeden A, McEver RP (2004). Surface fucosylation of human cord blood cells augments binding to P-selectin and E-selectin and enhances engraftment in bone marrow. *Blood*.

[78] Weber GF, Bronson RT, Ilagan J, Cantor H, Schmits R, Mak TW (2002). Absence of the CD44 gene prevents sarcoma metastasis. *Cancer Research*.

[79] Herrera MB, Bussolati B, Bruno S (2007). Exogenous mesenchymal stem cells localize to the kidney by means of CD44 following acute tubular injury. *Kidney International*.

[80] De Becker A, Van Hummelen P, Bakkus M (2007). Migration of culture-expanded human mesenchymal stem cells through bone marrow endothelium is regulated by matrix metalloproteinase-2 and tissue inhibitor of metalloproteinase-3. *Haematologica*.

[81] Itoh Y, Takamura A, Ito N (2001). Homophilic complex formation of MT1-MMP facilitates proMMP-2 activation on the cell surface and promotes tumor cell invasion. *The EMBO Journal*.

[82] Korinek V, Barker N, Moerer P (1998). Depletion of epithelial stem-cell compartments in the small intestine of mice lacking Tcf-4. *Nature Genetics*.

[83] Reya T, Duncan AW, Ailles L (2003). A role for Wnt signalling in self-renewal of haematopoietic stem cells. *Nature*.

[84] Neth P, Ciccarella M, Egea V, Hoelters J, Jochum M, Ries C (2006). Wnt signaling regulates the invasion capacity of human mesenchymal stem cells. *Stem Cells*.

[85] De Boer J, Wang HJ, Van Blitterswijk C (2004). Effects of Wnt signaling on proliferation and differentiation of human mesenchymal stem cells. *Tissue Engineering*.

[86] Neuss S, Becher E, Wöltje M, Tietze L, Jahnen-Dechent W (2004). Functional expression of HGF and HGF receptor/c-met in adult human mesenchymal stem cells suggests a role in cell mobilization, tissue repair, and wound healing. *Stem Cells*.

